# Splicing dysregulation as a driver of breast cancer

**DOI:** 10.1530/ERC-18-0068

**Published:** 2018-05-30

**Authors:** Abigail Read, Rachael Natrajan

**Affiliations:** 1 The Breast Cancer Now Toby Robins Research CentreThe Institute of Cancer Research, London, UK; 2 Division of Molecular PathologyThe Institute of Cancer Research, London, UK

**Keywords:** splicing, breast, molecular genetics

## Abstract

Breast cancer is known to be a heterogeneous disease driven by a large repertoire of molecular abnormalities, which contribute to its diverse clinical behaviour. Despite the success of targeted therapy approaches for breast cancer patient management, there is still a lack of the molecular understanding of aggressive forms of the disease and clinical management of these patients remains difficult. The advent of high-throughput sequencing technologies has paved the way for a more complete understanding of the molecular make-up of the breast cancer genome. As such, it is becoming apparent that disruption of canonical splicing within breast cancer governs its clinical progression. In this review, we discuss the role of dysregulation of spliceosomal component genes and associated factors in the progression of breast cancer, their role in therapy resistance and the use of quantitative isoform expression as potential prognostic and predictive biomarkers with a particular focus on oestrogen receptor-positive breast cancer.

## Introduction

**Figure 1:**
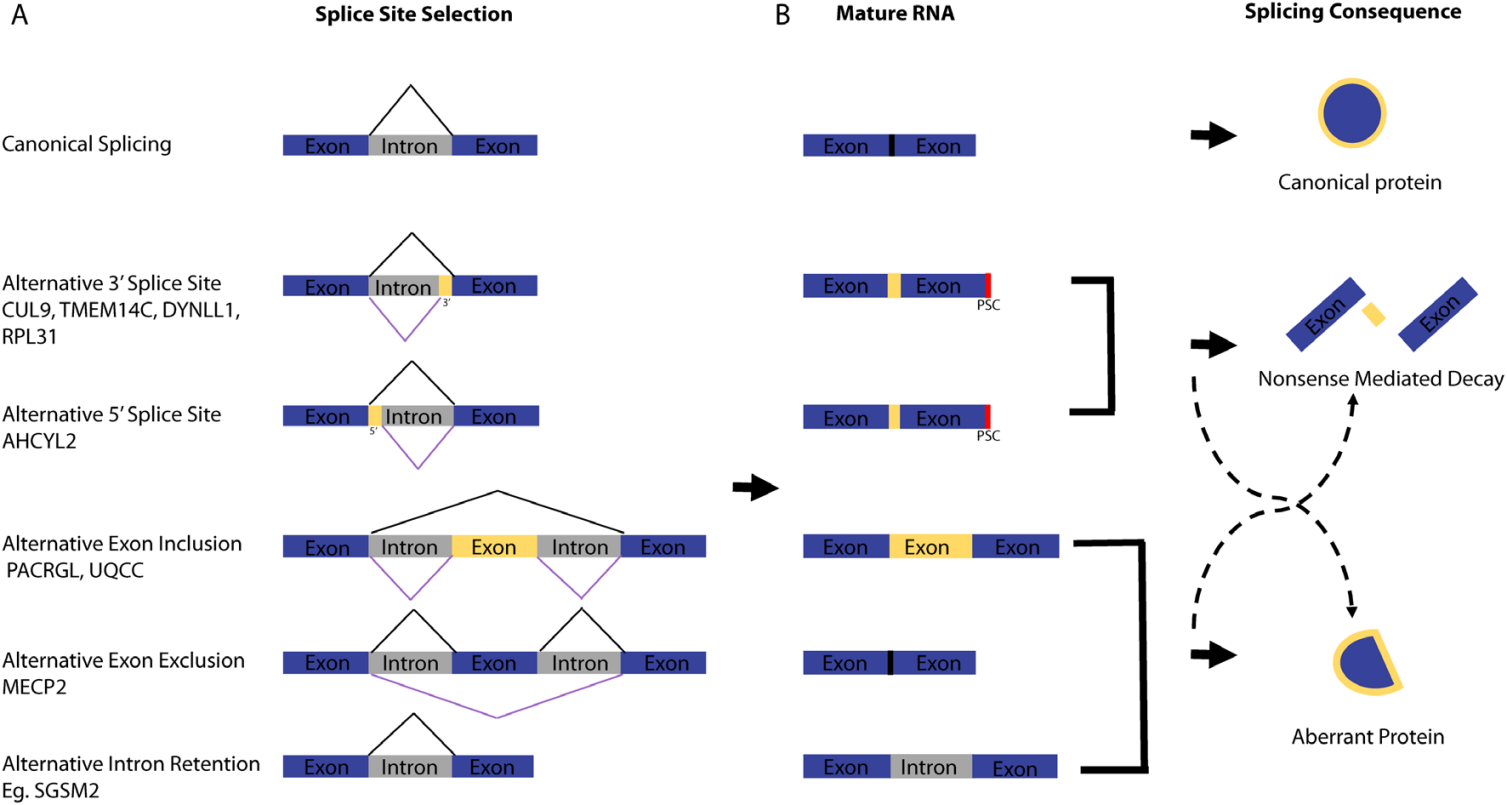
Mechanisms of alternative splicing in cancer. (A) Schematic of the possible ways in which alternative splicing can change the mRNA product. The product of canonical splicing is shown as well as the products of alternative splicing. Yellow represents non-canonical areas of the mRNA that are present in alternatively spliced transcripts. The black lines above the mRNA show where canonical splice sites are selected and the purple lines below the mRNA show where alternative splice sites are selected. Examples of genes for each event were obtained from (Darman* et al*. 2015). (B) The most likely product of the mRNA is indicated with solid dark arrows and the less likely but still possible products are indicated with dashed black arrows.

Dysregulation of alternative splicing (AS) is widely considered a new hallmark of cancer and its products are being acknowledged as potentially useful biomarkers (Ladomery 2013). Canonical RNA splicing takes place in all mammalian cells and during this process, pre-mRNA becomes mature mRNA via the excision of introns and pasting together of exons (Fig. 1). AS affects about 90% of human genes resulting in a diverse selection of isoforms from one gene, each having different structural and functional properties that lead to a larger and more diverse cellular proteome. Indeed throughout evolution, AS has been used to propel species development evidenced by an increase in AS in higher eukaryotes compared to lower (Keren* et al*. 2010).

Splicing is performed by the spliceosome, which is a multi-protein complex called a ‘metalloribozyme’ that is made up of five small nuclear riboproteins (snRNPs) that contain snRNAs and a large number of accessory proteins to recognise the pre-mRNA being spliced. Assembly of this complex takes place during transcription suggesting that transcription and splicing machineries are space restricted as they happen closely in time (Herzel* et al*. 2017). The most commonly occurring spliceosome is the U2-dependent spliceosome that is assembled from the U1, U2, U5 and U4/U6 snRNPs and is responsible for the splicing of 99% of human introns as reviewed by Dvinge* et al*. (2016). Splicing is a two-step reaction involving transesterification occurring between two RNA nucleotides. The spliceosome recognises introns containing the consecutive nucleotides GU at the 5′ splice site (SS) by U1 snRNP binding and an AG sequence at the 3′ SS by U2AF1 binding. In order to properly position the splicing machinery a key adenine (also referred to as the branch point (BP)) must be recognised by the splicing factor 1 protein (SF1) as well as recognition of the polypyrimidine tract (poly-Y) by the U2 small nuclear RNA auxiliary factor 2 (U2AF2) (Pandya-Jones 2011). When the spliceosome complex is correctly bound to the mRNA, it can carry out intron excision (Fig. 1A). Exons are subsequently joined together and release a lariat intron, which is then degraded. The spliceosome components are then released and recycled for use in subsequent rounds of splicing. Splicing factors such as serine/arginine-rich (SR) proteins (SRSF) and the splicing factor 3b complex (SF3B) work in association with the splicing core complex to coordinate canonical and AS. The expression levels and binding affinities of the different splicing factors play a stoichiometric role in determining the final isoform of the protein that is to be expressed (da Luz* et al*. 2017).

Dysregulation of the normal splicing process governs many aspects of cancer cell biology such as managing cellular proliferation, angiogenesis, resisting apoptosis, adapting cell metabolism, enhancing the ability to invade and metastasise and plays a role in resistance to cancer therapy (David & Manley 2010, Lee & Abdel-Wahab 2016). The role of AS in disease can result from aberrant splicing of a gene due to incorrect 5′ or 3′ SS recognition leading to intron retention, exon skipping or exon inclusion (Fig. 1A). AS may then lead to a premature stop codon resulting from a frame-shift, whereby these transcripts are subsequently degraded by nonsense-mediated decay (NMD) (Fig. 1B). There are multiple ways in which a cancer cell can induce aberrant splicing including: (1) when there is a mutation in the exon or surrounding introns that compromises the canonical splicing signal thereby allowing an alternative signal to dominate and an aberrant mRNA to be made; (2) a mutation in one of the splicing regulators interrupts SS selection and results in a pattern of AS in multiple genes; (3) changes in histone acetylation of alternative exons (Khan* et al*. 2014) and (4) alterations in other RNA-binding proteins, splicing enhancers and suppressors or lncRNAs. Such splicing errors can lead to alterations in relative isoform expression of a particular mRNA or lead to an aberrant protein that has a change of function. A more detailed discussion on points 1 and 3 are detailed elsewhere (Martinez-Montiel* et al*. 2017). Aberrantly spliced apoptotic genes such as the RNA-binding protein *RBM5* have been implicated in breast cancers as having an opposing role because the resulting isoform is more anti-apoptotic (Fushimi* et al*. 2008). Another example is the B-cell lymphoma gene, *Bcl-x*, which can be spliced into two different isoforms, long and short*. Bcl-x(L)* has anti-apoptotic properties whereas *Bcl-x(s)* has pro-apoptotic properties. High levels of *Bcl-x(L*) are seen in various types of cancer (Boise* et al*. 1993, Takehara* et al*. 2001, Fushimi* et al*. 2008). A similar situation is seen with the myeloid cell leukaemia-1 gene and its two isoforms MCL-1(S) and MCL-1(L). The long isoform is anti-apoptotic and seen frequently increased compared to the short isoform in breast and ovarian cancer cells and is linked to gene amplification of *MCL-1* itself (Bae* et al*. 2000, Bingle* et al*. 2000, Gautrey & Tyson-Capper 2012). The choice between the long and short isoform is influenced by the splicing factors SRSF1 and SRSF5, which are also frequently upregulated in breast cancer (Gautrey & Tyson-Capper 2012).

Managing key cellular processes such as epithelial-to-mesenchymal differentiation (EMT) is a clear advantage of being able to manipulate the expression of different isoforms of a certain gene (Shapiro* et al*. 2011). As such, acquiring the ability to hijack these processes is critical in the evolution of a cancer cell in order to provide a fitness advantage. Given this, it is reasonable to postulate that the characterisation of the splicing programme of a cancer cell could predict its genomic and mutational status and potentially treatment outcome (Danan-Gotthold* et al*. 2015). Indeed, differential expression of AS transcripts in specific subtypes of breast cancer may add additional prognostic information in addition to canonical gene expression or protein expression biomarkers.

## Evidence of splicing dysregulation in breast cancer

Since the seminal studies from Perou and colleagues describing the intrinsic subtypes of breast cancer (Perou* et al*. 2000), it is now widely accepted that the molecular make-up of breast cancer is heterogeneous and governed by differences in transcriptional make up. Inevitably, this also applies to the degree of isoform usage in cancer cells as well. For instance, well-known driver oncogenes and tumour suppressor genes such as *ERBB2* and *BRCA1* are known to be differentially spliced in different subtypes of breast cancer (as reviewed by Martinez-Montiel* et al*. 2017). BRCA1, which is involved in homologous recombination DNA repair, is alternatively spliced in breast cancer to exclude exon 11 that contains the nuclear localisation signal (Thakur* et al*. 1997). The ∆11q isoform produces a protein that is absent from the nucleus and is therefore unable to assist in DNA damage repair. Studies have shown that downregulation of the full-length nuclear *BRCA1* isoform and overexpression of the cytoplasmic ∆11q isoform is evident in subsets of breast cancer and is potentially mediated through the presence of a non-functional TRA2β splicing factor (Raponi* et al*. 2014, Wiener* et al*. 2015). Another example is the ERBB2 tyrosine kinase signalling receptor, which is often found as alternatively spliced in breast cancer as the *∆16HER2* isoform. *∆16HER2* is constitutively active as a homodimer and promotes transformation in the mammary gland (Marchini* et al*. 2011). *BRCA1* and *ERBB2* splicing, as well as splicing of BCL-X and MCL-1 as described earlier, are examples of common driver oncogenes and tumour suppressor genes that can be aberrantly spliced in breast cancer. AS has also been shown to regulate protein diversity of the oestrogen receptor itself. In particular, previous studies have shown the ERαΔ5 splice variant has a positive effect on activation of transcription in the absence of oestrogen leading to constitutive transcriptional activation (Fuqua* et al*. 1991, Bollig & Miksicek 2000). ESR1 aberrant splicing events have also been identified in circulating tumour cells from metastatic breast cancer patients that have progressed on endocrine therapy, suggesting a role in mediating resistance (Beije* et al*. 2018). Current data sets describing AS events in the context of spliceosomal gene mutations, however, do not show changes in splicing of the oestrogen receptor itself (Darman* et al*. 2015, Maguire* et al*. 2015). Alternatively spliced isoforms of genes known to be transcriptionally regulated by the oestrogen receptor such as Cyclin D1 (cyclin D1b) and FGFR1 (FGFR1-beta) are also associated with poor prognosis in ER+ breast cancer (Wei* et al*. 2011, Wendt* et al*. 2014).

## Alternatively-spliced transcripts as prognostic and predictive biomarkers in breast cancer

The recent advent of RNA-sequencing technologies has revolutionised our view of the molecular make up of breast cancer. These advances now allow accurate global quantification of the transcriptional isoform make-up in individual tumours rather than relative quantification that is based on microarray probe design. Indeed, a number of studies have shown that alternative isoform usage can be specific to different breast cancer molecular subtypes (Menon* et al*. 2014, Sebestyen* et al*. 2015, Zhao* et al*. 2016, Gracio* et al*. 2017, Stricker* et al*. 2017). For instance, Sebestyen* et al*. identified a specific 7 gene isoform signature that accurately identified basal-like breast cancers, including a number of known driver genes such as *CTNND1* (Sebestyen* et al*. 2015). Analysis of the splicing balance (relative ratios of isoforms produced) in breast tumours revealed changes in isoform usage in oncogenic and tumour suppressive pathways that was not apparent when looking solely at gene expression data (Gracio* et al*. 2017). Importantly, it was found that the balance of different transcript isoforms was associated with patient prognosis. A subset of genes including the proto-oncogene *MYB* were identified to correlate with basal-like breast cancer patient survival based on varying isoform levels but not on whole gene expression analyses (Gracio* et al*. 2017). Additionally, splicing but not gene expression levels of immune-related genes *CCR7* and *FCRL3* were found to determine the immune control of the tumour. This has potential relevance given the role of lymphocytic infiltration in prognosis in breast cancer. Differential isoform usage can also stratify between different molecular subtypes of breast cancer. Indeed, global dysregulation of splicing specific to individual subtypes may drive the heterogeneous nature of breast cancer due to variation in the cellular proteome. Stricker* et al*. (2017) looked at the global isoform differences between ER+ and triple-negative breast cancer (TNBC) and identified a signature of subtype-specific alternatively spliced transcripts. Interestingly, around 63% of the genes that were found to be differentially expressed, between subtypes were also alternatively spliced. The particular type of splicing that occurred between the subtypes (exon skipping, intron retention, alternative acceptor or donor), however, was not significantly different indicating the unique splicing programmes of each intrinsic subtype is not necessarily due to the activity of one general splicing mechanism but more likely due to target gene selection (Stricker* et al*. 2017). Interestingly, this study also identified a significant difference in the total expression of some spliceosomal component genes themselves, such as *YBX1* and* MAGOH* suggesting dysregulation of spliceosomal component proteins governs splicing dysregulation.

Although clear differences in transcript isoforms have been identified in different molecular subtypes of breast cancer, to date, no study has assessed the value of alternatively spliced transcripts as prognostic and predictive clinical biomarkers for patient stratification and of treatment response to both standard chemotherapy and targeted endocrine therapy. Assessment of differences in transcript isoform expression could add much needed biomarkers for patients who are most likely to relapse on standard-of-care therapy. Ideally, this would need to be tested in the context of randomised clinical trial cohorts, where good-quality RNA-sequencing data at sufficient depth are acquired.

## Dysregulation of spliceosomal factors in breast cancer

Molecular alterations affecting spliceosomal component genes themselves are also known to be involved in breast cancer tumourigenesis. There is evidence that mutations, copy number alterations and differential expression of spliceosomal component genes and their interacting proteins are associated with specific molecular and histological subtypes of breast cancer as well as being associated with aggressive disease and resistance to therapy in multiple tumour types (Stark* et al*. 2009, Ng* et al*. 2012, Sotillo* et al*. 2015, Siegfried & Karni 2017). These alterations are thought to drive breast cancer progression through specific or novel isoform selectivity of key genes (Vanharanta* et al*. 2014, Anczukow* et al*. 2015, Gokmen-Polar* et al*. 2015, Maguire* et al*. 2015, Silipo* et al*. 2015, da Luz* et al*. 2017, Martinez-Montiel* et al*. 2017).

### Mutations in spliceosomal component genes

Mutations affecting different components of the spliceosome have been identified in a range of solid and non-solid malignancies (Papaemmanuil* et al*. 2011, Quesada* et al*. 2011, Biankin* et al*. 2012, Furney* et al*. 2013, Yoshida & Ogawa 2014). Mutations in the splicing factor SF3B1 are the most common across multiple tumour types, and are found at particularly high frequencies in myelodysplastic syndrome (MDS), chronic myeloid leukaemia (CLL), uveal melanoma (UV), pancreatic cancer and breast cancer. Mutations generally cluster at hotspot amino acid residues K700, R625, K666 and H662 (Cerami* et al*. 2012, Gao* et al*. 2013). However, each cancer type harbours a different variation of hotspot mutations. For example, K700E mutations are invariably found in breast cancer, pancreatic cancer and CLL, whereas UV and endometrial cancers harbour the R625, R666 and R662 hotspots, suggesting some tissue specificity of the mutations. SF3B1 hotspot mutations in CLL are associated with a poor prognosis. However, in UV and MDS the prognosis is better with the presence of an SF3B1 mutation (Quesada* et al*. 2011, Furney* et al*. 2013). Interestingly, additional spliceosomal component genes are also recurrently mutated at high frequencies particularly in MDS, including U2AF1, which has a distinct S34F/Y hotspot mutation and mutations in SRSF2 that are associated with a poor outcome in MDS (Thol* et al*. 2012) and ZRSR2. Both SRSF2 and ZRSR2 harbour mutations spread throughout the gene, suggestive of a tumour suppressive function (Yoshida* et al*. 2011). Mutations in these genes including SF3B1 occur in a mutually exclusive manner in MDS, suggesting that cells may tolerate only a partial deviation from normal splicing activity. Indeed, these genes are all involved in the 3′-SS recognition during pre-mRNA processing, inducing abnormal RNA splicing and compromised haematopoiesis (Yoshida* et al*. 2011), implicating splicing dysregulation as a major driving force behind the development of MDS.

**Figure 2:**
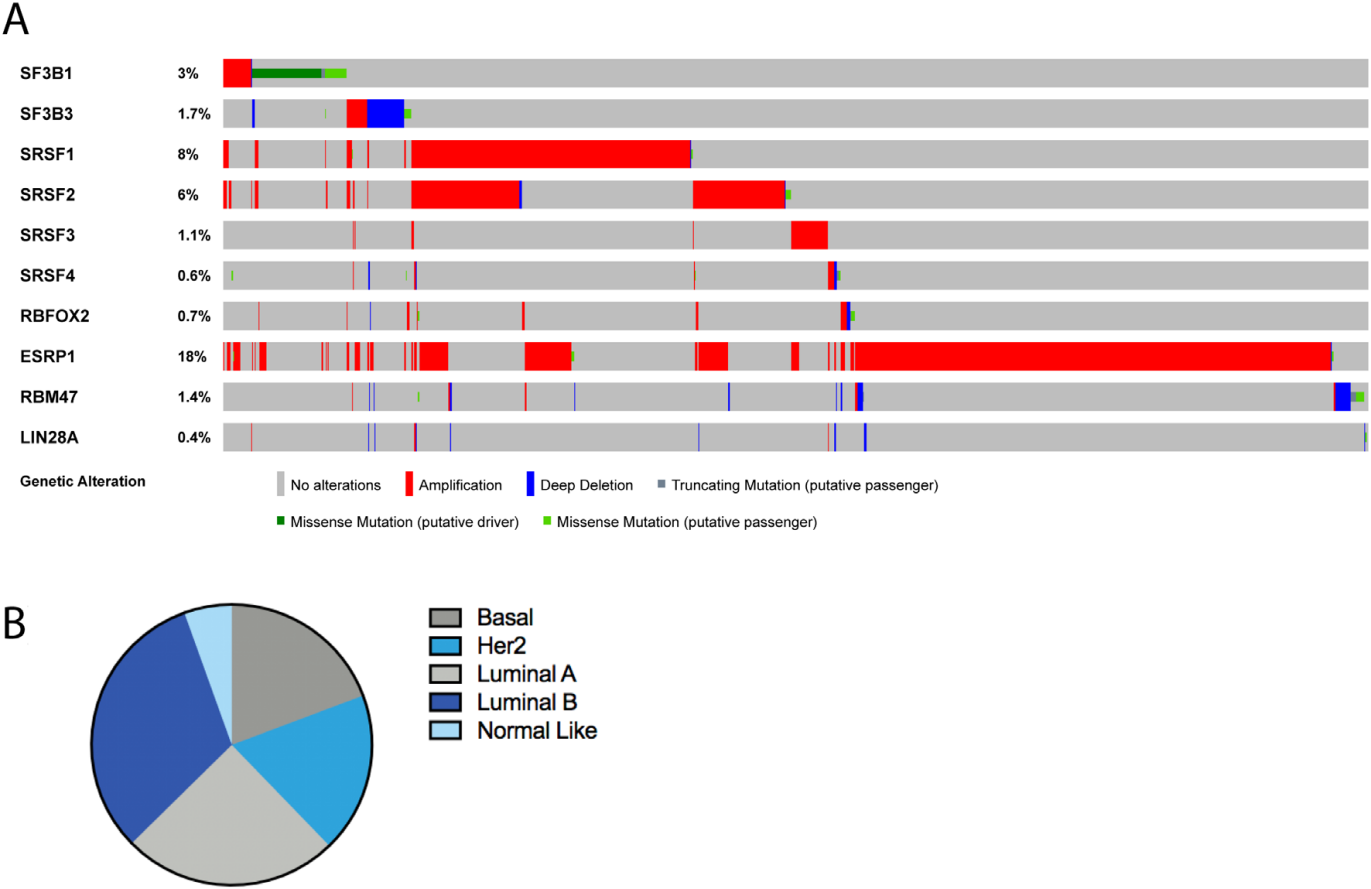
Summary of spliceosomal gene alterations in breast cancer. (A) cBioportal analysis of alterations in spliceosomal component genes from all available breast cancer data sets (Cerami* et al*. 2012, Gao* et al*. 2013). (B) Breakdown of patients with alterations by subtype from METABRIC and TCGA data with available PAM50 subtype calls. Basal = 19.3%, Her2 = 18.5%, Luminal A = 24.9%, Luminal B = 31.9%, Normal like = 5.5%.

Our group has explored the mutational repertoire of spliceosomal component genes in breast cancer from a meta-analysis of whole genome and exome sequencing data (Maguire* et al*. 2015) (Fig. 2). This analysis identified that around 5.6% of unselected breast cancers have mutations in spliceosome component genes at low frequencies. The most common spliceosomal gene mutation is *SF3B1*, which is associated with ER+ breast cancer and seen in around 3% of ER+ tumours (Pereira* et al*. 2016), whereas mutations in *SON* and *SAP130* appear to be associated with ER− disease (Maguire* et al*. 2015). Interestingly, we identified SF3B1 K700E mutations at higher frequencies in some rarer histological subtypes of breast cancer including 16% of papillary carcinomas and 8% of mucinous carcinomas of the breast, suggesting they may underpin their biology (Maguire* et al*. 2015). SF3B1 K700E mutations were also found to associate with losses of 16q11-q13 and gains of 16q12-q13 indicating a distinct mechanism of breast cancer progression independent of the canonical early event of 1q gain and 16q loss (Maguire* et al*. 2015).

The association of *SF3B1* mutations and breast cancer clinical prognosis, however, is unclear, although mutations are being increasingly seen in metastatic disease (Lefebvre* et al*. 2016, Pereira* et al*. 2016). Further studies however are needed in order to truly assess the effect of *SF3B1* hotspot mutations on outcome. Of note, *SF3B1* mutations have been observed in adenoid cystic carcinomas of the breast (an ER-negative special histological subtype) that has an excellent clinical outcome and at increased frequency in ER+ mucinous and papillary carcinomas of the breast. These data perhaps suggest that *SF3B1* mutations maybe associated with a good prognosis (Maguire* et al*. 2015, Martelotto* et al*. 2015).

SF3B is a complex that is part of the U2 spliceosome and controls 3′ SS recognition. Its core is required for alignment of the branch site proteins, which allows for correct branch site selection during the splicing process (Cretu* et al*. 2016). *SF3B1* (*SF3B155*) is the largest component of the SF3B complex and contains the HEAT superhelix domain consisting of 20 tandem repeats of two alpha helices joined by a short loop. Mutations in the HEAT domains, which are responsible for interacting with pre-mRNA and other pre-mRNA-binding proteins, result in a change in the tertiary structure that causes the selection of an alternative branch site (Darman* et al*. 2015, Alsafadi* et al*. 2016, Kesarwani* et al*. 2017). It is not known, however, whether the SF3B1 mutant protein has a stronger affinity for the newly exposed BP sequence or if it is coping with a disruption in binding to the canonical BP sequence (Darman* et al*. 2015). Indeed, mutations in SF3B1 lead to alternative branchpoint usage and subsequent usage of a 3′ cryptic SS. This leads to aberrant transcript expression and subsequent NMD of around half the aberrant transcripts and hence leads to protein downregulation (Darman* et al*. 2015, Alsafadi* et al*. 2016, Kesarwani* et al*. 2017).

Although present as hotspot single amino acid changes, SF3B1 mutations are thought to lead to a change in function. This is because knockdown or overexpression of the mutant protein does not recapitulate the aberrant splice pattern seen in mutant vs WT patients (Alsafadi* et al*. 2016). Additional evidence suggests that these mutations may actually be loss of canonical function. For instance, using the Degron-knock-in approach to inactivate mutant or WT alleles specifically, Zhou* et al*. found that degradation of only the mutant SF3B1 allele in heterozygous SF3B1-mutant cells had no effect on growth, whereas degradation of only the WT allele resulted in a decrease in viability of the cells (Zhou* et al*. 2015). This suggests that SF3B1 is not likely to be haploinsufficient given the cells are solely relying on the WT copy of the gene to survive. This observation helps explain why *SF3B1* mutations are uniformly heterozygous, as two copies of the mutant allele would likely be lethal.

The most common SF3B1 mutation in breast cancer is the K700E variant akin to CLL but K666Q and K666E are also observed, albeit at much lower frequencies (Maguire* et al*. 2015). Gene expression analysis in ER-positive disease shows that SF3B1 mutations affect regulators of the cell cycle, metabolism and motility as well as protein degradation and apoptosis, and splicing regulation itself (Maguire* et al*. 2015). Commonly differentially spliced mRNAs have been associated with SF3B1 mutations across tumour types including UV, chronic lymphocytic leukaemia, pancreatic cancer and breast cancer. Although a large number of transcripts have been identified to be aberrantly spliced and some are cancer specific (e.g. ABCB7 AS is only observed in MDS and gives rise to increased mitochondrial iron accumulation found in MDS patients with ring sideroblasts (Dolatshad* et al*. 2016), the overlap is rather strikingly consistent between tumour types, suggesting that there is a distinct signature of genes that are alternatively spliced and furthermore can be used as markers of the mutation status (Quesada* et al*. 2011, Biankin* et al*. 2012, Furney* et al*. 2013, Maguire* et al*. 2015, Dolatshad* et al*. 2016, Obeng* et al*. 2016, Wang* et al*. 2016). However, it has not yet been identified which of the many differentially spliced genes is/are responsible for the tumorigenic phenotype and if these are different between different cancer types. In our study, we used siRNA to silence different genes that had been identified as alternatively spliced in our data set as well as across multiple cancer types. Silencing eight different genes (*ABCC5*, *ANKHD1*, *DYNLL1*, *F8*, *RPL31*, *TMEM14C*, *UQCC* and *CRNDE*) did not show any changes in viability (Maguire* et al*. 2015). Given around half of all aberrantly expressed transcripts are subjected to NMD, they could be acting as tumour suppressors rather than in an oncogenic manner and will need to be explored in the future.

### Spliceosomal component genes as oncoproteins in breast cancer

As well as mutations, alterations in components of the spliceosome, such as deletions or amplifications, are commonly seen across breast cancer (Fig. 2; Tables 1 and 2). In a similar vein to spliceosomal component mutations, they may lead to dysregulation of canonical splicing. SF3B3 (SF3B130) a component of the SF3B complex has been found to be significantly overexpressed in ER+ breast cancers and is associated with aggressive disease and resistance to tamoxifen therapy (Gokmen-Polar* et al*. 2015). SF3B3 is positioned closely to SF3B1 in the U2 complex and helps maintain the HEAT domain’s structural plasticity and has the ability to alter pre-mRNA splicing hence affecting gene expression in the cell (Garcia-Blanco* et al*. 2004). Overexpression of SF3B3 has thus been postulated to contribute to spicing aberrations in cancer cells. In clear cell renal cell carcinoma, SF3B3 overexpression was found to increase the expression of the pro-proliferative full-length isoform of *EZH2* and not the commonly expressed *EZH2∆14* that is found in normal tissue (Chen* et al*. 2017), thus promoting tumourigenicity *in vivo*. It could be that EZH2 AS plays a role in mediating the aggressive behaviour in endocrine resistant ER+ breast cancer as well; however, this is yet to be elucidated. SF3B3 has also been found to be amplified and highly expressed at the transcript level in basal-like breast cancers (Srihari* et al*. 2016). Overall, the level is actually higher in ER− than ER+ disease, perhaps highlighting the higher proliferative rate of these tumours.

**Table 1:** Summary of spliceosome component genes and RNA-binding proteins found altered in breast cancer.

Splicing factor/RNA-binding protein	Gene name	Alteration	Occurrence in BrCa (%)	Functional impact
SF3B1	Splicing factor 3B subunit 1	Mutation and CNA	3	Change of function, oncogenic
SF3B3	Splicing factor 3B subunit 3	CNA	1.7	Oncogenic
SRSF1	Serine/arginine rich splicing factor 1	CNA	8	Oncogenic
SRSF2	Serine/arginine rich splicing factor 2	CNA	6	Oncogenic
SRSF3	Serine/arginine rich splicing factor 3	CNA	1.1	Oncogenic
SRSF4	Serine/arginine rich splicing factor 4	CNA	0.6	Oncogenic
RBFOX2	RNA-binding protein fox-1 homolog 2	CNA	0.7	EMT regulator
ESRP1	Epithelial splicing regulatory protein 1	CNA	18	EMT regulator
RBM47	RNA-binding motif protein 47	CNA	1.4	Downregulation
LIN28A	Lin-28 Homolog A	CNA	0.4	Loss of function

Sourced from all breast cancer studies available in cBioportal. *n* = 4587 sequenced cases.

CNA, copy number alteration.

The SRSF family of proteins are serine-arginine-rich splicing factors that are commonly found to be mutated or dysregulated in cancer (Das & Krainer 2014). These proteins contain RNA recognition motif (RPM) domains that contact the mRNA and also interact with other splicing machinery (Das & Krainer 2014). *SRSF1* also referred to as SF2/ASF is the most common protein of this family to play a role in breast cancer and overexpression is associated with a poor prognosis in ER+ breast cancers (Anczukow* et al*. 2012). Overexpressing SRSF1 in 3D mammary organotypic assays is associated with larger acini structures indicating its oncogenic phenotype (Anczukow* et al*. 2012). This study also highlighted specific isoform dysregulation of the tumour suppressors BIM and BIN1, which resulted in loss of their pro-apoptotic functions (Karni* et al*. 2007, Anczukow* et al*. 2012). SRSF1 upregulation is thought to play a role in EMT through AS modulation of its transcriptional target genes (Valacca* et al*. 2010). Mechanistically, this is linked back to the splicing regulator Sam68, which modulates levels of SRSF1 (Valacca* et al*. 2010). It was found that SRSF1 is more likely to facilitate exon inclusion when it binds closer to the 5′ site of the splice junction and promotes exon skipping or inclusion when it binds to the 3′ end (Anczukow* et al*. 2015). SRSF1 was found to alternatively splice *CASC4* by including exon 9, resulting in a longer protein. When tested alone, overexpression of this isoform of *CASC4* phenocopied the tumorigenic abilities of SRSF1 overexpression by increasing proliferation and acinar size and decreasing apoptosis (Anczukow* et al*. 2015). These data highlight promising targets for therapeutic development in patients with SRSF1 overexpression.[Table tbl2]

**Table 2 tbl2:** Number and percentage of patients pertaining to each subtype with an alteration in the specified spliceosome component genes.

	SF3B1	SF3B3	SRSF1	SRSF2	SRSF3	SRSF4	RBFOX2	ESRP1	RBM47	LIN28A
Basal*n* = 391	12 (3.07)	10 (2.56)	11 (2.81)	22 (5.63)	15 (3.84)	6 (1.53)	5 (1.28)	84 (21.48)	13 (3.32)	1 (0.26)
Her2*n* = 287	9 (3.14)	3 (1.05)	39 (13.59)	25 (8.71)	2 (0.70)	1 (0.35)	2 (0.70)	99 (34.49)	6 (2.09)	1 (0.35)
Luminal A*n* = 909	40 (4.40)	17 (1.87)	31 (3.41)	26 (2.86)	5 (0.55)	2 (0.22)	2 (0.22)	103 (11.33)	5 (0.55)	3 (0.33)
Luminal B*n* = 590	19 (3.22)	5 (0.85)	99 (16.78)	50 (8.47)	3 (0.51)	2 (0.34)	4 (0.68)	159 (26.95)	2 (0.34)	1 (0.17)
Normal like*n* = 179	4 (2.23)	3 (1.68)	10 (5.59)	7 (3.91)	0 (0.00)	1 (0.56)	0 (0.00)	23 (12.85)	1 (0.56)	0 (0.00)

Data were derived from METABRIC and TCGA samples with available PAM50 subtype scores (*n* = 2363). Percentages in brackets.

Other members of the SRSF family have also been implicated in breast cancer. For instance, *SRSF2* gene amplification at 17q25 has been observed in 6% of breast cancers, although it is uncertain whether this plays an oncogenic role, given evidence that mutations are loss of function in this gene (Chung* et al*. 2017). Finally, SRSF4 overexpression has been identified in a small subset of breast cancer and its expression has been found responsible for cisplatin-induced AS that leads to apoptosis. Experiments where SRSF4 was silenced showed a decrease in apoptosis upon treatment with cisplatin and highlight the possibility of modulating splicing to regulate chemotherapy sensitivity (Gabriel* et al*. 2015).

### Dysregulation of spliceosomal accessory proteins

Along with the major components of the spliceosome that were described earlier, there are also other regulators of splicing that have been found to be mutated or dysregulated in breast cancer. LIN28A has been identified specifically in HER2-positive breast cancer as being a regulator of AS through interactions with hnRNPA1 (Yang* et al*. 2015, Xiong* et al*. 2017). Loss of LIN28A in breast cancer results in isoform switching of the ENAH gene, which is overexpressed in some primary breast tumours (Yang* et al*. 2015, Xiong* et al*. 2017). It has also been identified as a feature of the malignant phenotype in a model of breast cancer progression and has been correlated with an unfavourable outcome in HER2-positive breast cancer (Du* et al*. 2012). Other examples are the epithelial splicing regulatory proteins (ESRP1 and ESRP2), which are splicing factors that have been found to regulate the AS that governs EMT and are amplified in breast cancers (Warzecha* et al*. 2009, Brown* et al*. 2011, Bebee* et al*. 2015) and regulate EMT in breast tumours by activating AKT signalling (Brown* et al*. 2011). The RNA-binding protein RBFOX2 is also involved in cellular transition, whose upregulation can perturb splicing events in breast cancer (Du* et al*. 2012, Lapuk* et al*. 2010). During EMT, RBFOX2-regulated splicing shifts from EMT-specific events, subsequently leading to a higher degree of tissue invasiveness (Braeutigam* et al*. 2014). Another RNA-binding protein, RBM47, has the ability to alter splicing by binding to introns and 3′ UTRs and loss of expression has been shown to prevent breast cancer progression and metastasis (Vanharanta* et al*. 2014). Taken together, these lines of evidence point to a fundamental role triggered by splicing dysregulation in breast cancer cells that can cause detrimental effects and lead to the progression of disease.

## Evidence of oncogene-induced dependency on the spliceosome

Aside from alterations in spliceosomal component genes themselves, there is emerging evidence that oncogene activation imparts a functional dependency on SF3B1 and other components in breast cancer. A number of spliceosomal component proteins are known transcriptional targets of the oncoprotein MYC (including *SF3B1* and *SRSF1*) and have been shown to both contribute to and cooperate with MYC in malignant transformation (Das* et al*. 2012, Koh* et al*. 2015). For instance, MYC addicted TNBCs cells have been shown to impart a specific dependency on the spliceosome via *BUD31* and *SF3B1* (Hsu* et al*. 2015) and impaired tumourigenesis was observed when SF3B1 was knocked down or pharmacologically inhibited in breast cancer cells MYC hyperactivation (Hsu* et al*. 2015). This could be explained due to the increased burden put on the spliceosome when the rate of transcription is increased due to MYC signalling. Recently, knockdown of SF3B1 was found to result in apoptosis in TNBC with MCL-1 inactivation being a likely mechanistic explanation, given MCL-1 is a SF3B1 splicing target (Gao & Koide 2013, Sridhar *et al*. 2017). Interestingly, MYC and MCL-1 have been shown to cooperate in chemoresistant TNBCs (Lee* et al*. 2017). This could be further support for the intricate co-operation of MYC with the spliceosome and the resulting changes in isoform dominance that allow the manipulation of cancer cells. In addition, SRSF1 is a known direct target of MYC. MYC induction leads to SRSF1-mediated AS of key protein isoforms involved in proliferation and anchorage-independent growth such as MKNK2 and TEAD1 (Anczukow* et al*. 2012, Das* et al*. 2012), which is in part through potentiating eIF4E activation (Anczukow* et al*. 2012, Das* et al*. 2012). Together, these studies suggest that multiple spliceosomal proteins are critical MYC targets that contribute to its oncogenic potential by enabling MYC to regulate the expression of specific protein isoforms via AS.

## Therapeutic targeting of the spliceosome

There is emerging evidence that disruption of spliceosomal proteins induces selectivity to inhibitors that target the spliceosome. Indeed a number of these inhibitors have been developed including Spliceostatin A, Pladienolides (including E7107) and meyamycin analogues that are all specific SF3B inhibitors as reviewed in (Lee & Abdel-Wahab 2016) that inhibit canonical splicing (Kaida* et al*. 2007). We, and others, have shown that *SF3B1*-mutant cells are selectively sensitive to spliceosomal inhibitors (Maguire* et al*. 2015, Obeng* et al*. 2016). Moreover, SF3b inhibition in *SF3B1* mutant cells resulted in a change in the reversal of the conserved splicing signature, suggesting that *SF3B1* mutations are change of function rather than loss of function and that these alterations in aberrant isoforms could be used as biomarkers of therapeutic response (Maguire* et al*. 2015). There is additional evidence that other spliceosomal gene mutations can be therapeutically targeted with spliceosomal inhibitors. These include SRSF2 mutations, whereby genetically modified mice expressing the Srsf2(P95H) mutation, were sensitive to treatment with the spliceosome inhibitor E7107, which decreased leukaemic burden (Lee* et al*. 2016). Similar selective sensitivity in mutant U2AF1 cells to sudemycins has also been reported in *in vitro* and *in vivo* (Shirai *et al*. 2015). In addition, MYC-addicted TNBCs have been shown to be more sensitive to inhibition with the spliceosome inhibitor SD6 than MYC non-addicted cells are (Hsu* et al*. 2015), a mechanism that is likely due to the increased stress and dependency on SF3B1 (as discussed earlier). Further functional studies in the context of clear cell renal carcinoma show that knockdown of SF3B3 in SF3B3-overexpressing cells *in vivo* reduced tumour growth, highlighting the potential utility of SF3b inhibitors as a therapeutic agent for patients with SF3B3 amplification and/or overexpression (Chen* et al*. 2017). These lines of evidence raise the possible clinical utility of SF3b inhibitors in patients with additional spliceosomal gene mutations as well as other indirect reliance on the spliceosome. Further studies are warranted to ascertain if overexpression of spliceosomal genes also confers sensitivity to these compounds in breast cancer.

Phase one clinical trials have been performed for E7107 in patients with solid tumours and although the drug has been shown to be on target in patients (i.e. perturbs splicing), the US and European trials were suspended due to an unexpected toxicity involving bilateral optic neuritis (Eskens* et al*. 2013, Hong* et al*. 2014). Further studies to understand the causes of toxicity as well as new clinical trials will be necessary to take advantage of splicing’s therapeutic vulnerability in cancer. Currently, H3 biomedicine is testing the compound H3B-8800, which inhibits the SF3b complex and was successful in preclinical studies treating a range of spliceosomal mutant cancers (Buonamici* et al*. 2016). The compound is now in phase one studies (Nbib2841540) for MDS, acute myeloid leukaemia and chronic myelomonocytic leukaemia.

## Conclusions

Mutations and changes in expression of splicing factors that lead to aberrant splicing is a hallmark of cancer that is also relevant to breast cancer. Development of prognostic and predictive aberrant splicing signatures specifically to predict patients that will respond to endocrine (or indeed CDK4/6 inhibitor) therapy could be particularly useful going forward. The increasing technical advances in sequencing methodologies, particularly those that aim to increase RNA read lengths, will undoubtedly enhance the ability to detect these events in the future and further increase our understanding of aberrant transcript expression on breast cancer tumourigenesis and therapy resistance. There is increasing evidence that spliceosomal component genes themselves are dysregulated in breast cancer, through mutations in *SF3B1* that are also observed in metastatic disease and upregulation of SF3B3 and SRSF1 in particular, which are associated with resistance to endocrine therapy. Dissecting the function of the expression of the consequent alternatively spliced transcripts would give insight into the mechanism of these alterations and the role they play in therapy resistance. Indeed with the development of spliceosome inhibitors themselves, and exciting preclinical data in other tumour types highlight a potential novel treatment strategy in combination with endocrine therapy and CDK4/6 inhibitors for patients with metastatic disease with spliceosomal gene alterations.

## Declaration of interest

The authors declare that there is no conflict of interest that could be perceived as prejudicing the impartiality of this review.

## Funding

The authors thank Breast Cancer Now for funding this work as part of Programme Funding to the Breast Cancer Now Toby Robins Research Centre.
